# Profile of the 2016 dengue outbreak in Nepal

**DOI:** 10.1186/s13104-018-3514-3

**Published:** 2018-07-03

**Authors:** Ramawatar Prasad Khetan, David A. Stein, Santosh Kumar Chaudhary, Ramanuj Rauniyar, Bishnu Prasad Upadhyay, Umesh Prasad Gupta, Birendra Prasad Gupta

**Affiliations:** 1 MB Kedia Dental College Private Limited, Birgunj, Tribhuvan University, Kathmandu, Nepal; 2 Department of Biomedical Sciences, Oregon State University, Corvallis, OR USA; 3 Virology Unit, Central Department of Biotechnology, Tribhuvan University, Kathmandu, Nepal; 4 National Public Health Laboratory, Ministry of Health, Government of Nepal, Kathmandu, Nepal; 5 Central Diagnostic Laboratory and Research Centre Pvt. Ltd, Kathmandu, Nepal

**Keywords:** Dengue fever, Dengue virus serotype 1, Outbreak, Nepal

## Abstract

**Objective:**

The objective of this study was to obtain clinical, virological and demographic data detailing the 2016 dengue outbreak in Nepal.

**Results:**

Dengue disease was first reported in Nepal in 2004 and several major outbreaks have occurred since then, with a significant impact on public health. An outbreak of dengue fever occurred in Nepal during June to November 2016, with a peak number of cases reported in September. 1473 patients with laboratory confirmed DENV infections visited or were admitted to hospitals during this period. The most common clinical symptoms included fever, headache, joint pain and thrombocytopenia. Serotyping of 75 serum samples from patients having fever for less than 4 days was carried out with a dengue virus (DENV) serotype-specific RT-PCR strategy. Our results indicate that the dengue outbreak in Nepal during 2016 was caused predominantly, if not exclusively, by DENV-1, representing a shift in the prevailing serotype from DENV-2, the dominant serotype characterizing the 2013 dengue epidemic in Nepal. Hopefully, this report will assist Nepalese public health agencies in developing improved dengue-related programs including mosquito-vector control, DENV surveillance, and diagnosis and treatment of dengue fever patients, in order to reduce the impact of future dengue epidemics.

**Electronic supplementary material:**

The online version of this article (10.1186/s13104-018-3514-3) contains supplementary material, which is available to authorized users.

## Introduction

Dengue virus (DENV) is a species in the genus *Flavivirus* of the family *Flaviviridae* and exists as numerous genetically distinct strains, each of which can be categorized into one of the four serotypes: DENV-1, DENV-2, DENV-3, and DENV-4 [1]. DENV is a single stranded, positive sense RNA virus, approximately 11 kb long. The genome codes for three structural proteins-nucleocapsid or core (C), a membrane-associated (M), an enveloped (E) glycoprotein, and seven non-structural (NS) proteins (NS1, NS2A, NS2B, NS3, NS4A, NS4B and NS5). DENV is transmitted to humans primarily by *Aedes aegypti* mosquitos and is the causative agent of dengue disease [2].

DENV occurs in tropical and subtropical regions worldwide and is the most common and widespread arboviral infection of humans. DENV infections typically result in tens of millions of clinical cases of dengue disease yearly, causing an enormous health, social and economic burden, mostly in low- and middle-income countries [3] Until recently, dengue disease was generally classified as dengue fever (DF), dengue hemorrhagic fever (DHF) or dengue shock syndrome (DSS). However, in 2009, The World Health Organization (WHO) [4] revised the guidelines for dengue disease classification. Cases of clinical infection are now classified as mild, moderate or severe dengue disease [5]. Mild or moderate dengue disease are considered non-life-threatening whereas severe dengue disease (associated with severe plasma leakage, severe bleeding, or organ failure) is considered a life-threatening condition (previously referred to as DHF and/or DSS). For the purposes of our study, we refer to mild or moderate dengue disease cases together as dengue fever (DF) to distinguish them from cases of severe dengue disease (SDD) [6]. Any symptomatic case of dengue disease is a miserable experience and results in several days or more of convalescence. It is generally accepted that a symptomatic initial DENV infection, with any single serotype, typically presents as mild or moderate dengue disease, but that subsequent infection with a DENV strain belonging to a different serotype is more likely to result in severe dengue disease, which can be fatal. The immunopathologic phenomenon in which non-neutralizing antibodies produced in response to an initial infection with one serotype remain in circulation and contribute to a more severe clinical outcome upon infection with a second serotype is known as antibody dependent enhancement (ADE) [7].

Nepal is a Himalayan country surrounded by the dengue-endemic countries India and China [8, 9]. In Nepal, the first case of dengue disease was reported in 2004 [10] followed by 32 laboratory confirmed cases during 2006. Virus sequencing of patient serum samples obtained during the 2006 outbreak showed that DENV strains from all four serotypes were circulating in the nine Terai regions of southern Nepal, however, no detailed epidemiologic data was collected [10, 11]. A small number of dengue disease cases were reported in 2007–2009 [10] followed by major outbreaks in 2010 and 2013 with the prevailing serotypes DENV-1 and DENV-2 respectively [12]. Another major dengue outbreak occurred in 2016, with clinical cases reported from several districts of Nepal. The aim of our study was investigate the characteristics and extent of the 2016 outbreak, including DENV serotype identification and demographic data associated with clinical cases.

## Main text

### Methods

Clinical information and blood samples were collected from patients who visited or were admitted to various clinics or hospitals during June–November 2016. Disease symptoms were diagnosed by clinicians and classified as dengue fever (DF) or severe dengue disease (SDD) based on the WHO classification (WHO 2009) [13]. Patients were suspected to have DF when presenting with acute onset of high fever lasting for 2–7 days and exhibiting at least two of the following symptoms: fever, rash, headache, retro-orbital pain and leucopenia. A patient was suspected of having SDD when hemorrhagic manifestations such as mucosal bleeding and plasma leakage were observed in addition to the features of DF described above. Patients suspected of having DF or SDD underwent further examinations for confirmation of the illness. A case was labeled as “probable” if the patient was found to harbor IgM antibodies specific against DENV or as “confirmed” when the presence DENV RNA was detected by RT-PCR.

Blood draws were collected from patients at the various clinical settings. Serum samples were obtained by centrifugation, and then assayed with the Panbio Dengue IgM capture ELISA diagnosis kit, for the detection of IgM antibodies against DENV, following protocols supplied with the product by the manufacturer. For detection of dengue NS1 antigen in the acute sera, the NS1 ELISA kit (Pan Bio, Australia) and its supplied protocols were used. For each sample, Panbio units were calculated according to the manufacturer’s instructions to determine if positive or negative for DENV.

Dengue viral RNA was assayed by reverse transcriptase PCR (RT-PCR) using primers designed to amplify a specific region of the Core-Pre-membrane (C-PrM) region of DENV genomic RNA. Briefly, RNA was isolated from 140 μl of each serum sample using the Nucleospin viral RNA isolation kit (MACHEREY–NAGEL, Germany) according to the manufacturer’s instructions. RT-PCR was then carried out using Access quick RT PCR Master mix (Promega, USA) with primers D1F and Dencom R2, for 40 cycles, as described previously (Additional file 1: Table S1) [14]. These primers target sequences which are highly conserved across the DENV virome and amplify a 654 bp product (corresponding to the sequence between nt 134–785) [14, 15]. A nested-primers PCR strategy was then used to distinguish the serotype identity of the amplicons. This method utilized a single forward primer (D1F) and series of reverse primers DNS1, DNS2, DNS3, and DNS4, which specifically distinguish DENV-1, DENV-2, DENV-3 and DENV-4 respectively (Additional file 1: Table S1) [16].

### Results

**Fig. 1:**
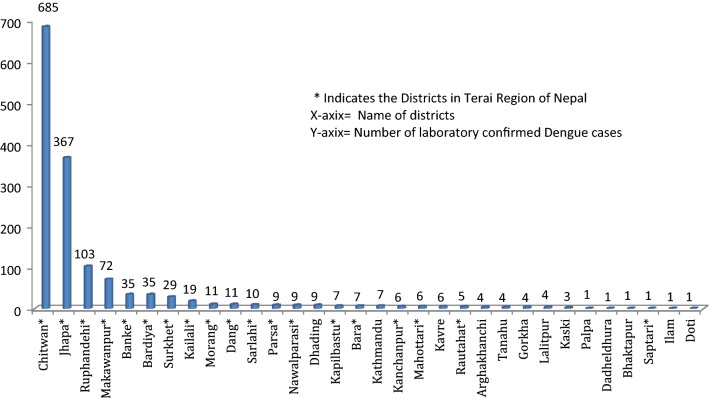
Incidence of dengue disease cases in Nepalese districts during 2016 outbreak

**Fig. 2:**
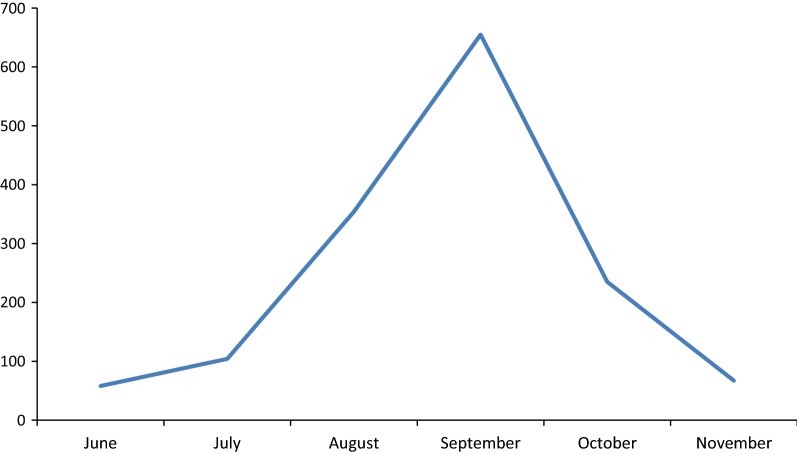
Monthly distribution of dengue disease cases during 2016 outbreak

The first significant outbreak of dengue in Nepal was reported in 2006 when around 35 cases occurred. In the years 2007–2009, 10–30 confirmed cases were reported each year. Major outbreaks occurred in 2010 and 2013 with 917 and 683 cases reported, respectively, while the intervening years of 2011–2012 had 79 and 183 cases respectively [17]. In 2016, 5769 persons with symptoms consistent with dengue disease visited various hospitals and clinics in Nepal. Among those, 1473 were dengue-positive based on laboratory confirmation representing a marked increase compared to previous years. Serotyping of 75 serum samples from patients having fever for < 4 days was carried out with a DENV (dengue virus)-serotype-specific reverse transcriptase polymerase chain (RT-PCR) strategy. The highest number of cases was in Chitwan district followed by other southern Nepal districts Jhapa, Rupandehi and Makwanpur; however clinical dengue cases were reported over a wide geographic area, in 32 of Nepal’s 75 total districts (Fig. 1). The major clinical features of patients who reported to the various clinical settings during this outbreak were fever (100%), headache (71.3%), rashes (11.3%), retro-orbital pain (23.5%), vomiting (23.4%), joint pain (32.1%) and thrombocytopenia (85.7%). Minor symptoms reported included abdominal pain and a feeling of restlessness. The infection was found to be at a higher in males in the 19–41 years age group with a child–adult ratio of 0.3:1 and male–female ratio of 6:4. The patients had a mean age (± standard deviation) of 37.85 (± 7.14) years. Clinical symptoms and serotype analysis of ten dengue disease patients with platelet counts of less than 50,000/mm^3^ are shown in Table 1. The outbreak peaked in September, corresponding to the tail end of monsoon season in Nepal, and subsided around the last week of November (Fig. 2).

**Table 1:** Symptoms and serotype of ten dengue disease patients with platelet count of less than 50,000/mm^3^

ID	Age (years)	Sex	Disease category	platelet count (× 1000/mm3)	Serotype	NS1 ELISA	Symptoms
1	22	M	DF	45	DENV-1	+	Fever, headache, joint pain
2	37	F	DF	40	DENV-1	+	Fever, headache, joint pain
3	33	M	DF	39	DENV-1	+	Fever, restlessness, vomiting
4	50	M	DF	32	DENV-1	+	Fever, headache, vomiting, joint-pain, restlessness
5	45	M	DF	45	DENV-1	+	Fever, headache restlessness, loss of appetite
6	24	M	DF	42	DENV-1	+	Fever, joint pain, loss of appetite
7	18	F	DF	30	DENV-1	+	Fever, restlessness, loss of appetite
8	36	M	DF	29	DENV-1	+	Fever, headache, nausea
9	28	M	DF	37	DENV-1	+	Fever, anorexia, nausea, fever, abdominal pain
10	52	F	DF	47	DENV-1	+	Fever, headache, muscular pain, nausea

### Discussion

Since the first reported endogenous cases in 2006, the number of dengue disease cases annually has been increasing overall in Nepal [18]. The geographical footprint of both the Ae. aegypti vector and dengue disease has expanded from the Terai lowland of southern Nepal and now includes the most populous urban area, Kathmandu, located in a highland region of central Nepal [19]. As observed in previous outbreaks as well, there was a sharp increase in both the number of mosquitos and number of dengue disease cases during and soon after the monsoon season of June–September in 2016. In this study, the majority of infected patients were of age 19–41 years, with a higher number of male than females represented, a pattern similar to that reported for previous outbreaks [20].

Dengue disease cases were most common in Chitwan and Jhapa districts, a region representing the commercial center of central-southern Nepal. As well, the Chitwan/Jhapa region is a transportation gateway to India and a hub for people travelling, primarily for economic reasons, between Nepal and India. Our data suggest that this region was likely the center of the 2016 outbreak and that DENV and dengue disease likely spread outward from there to other urban areas in Nepal. The spread of dengue disease from the Chitwan/Jhapa region to other parts of Nepal has been attributed to in part to ineffective vector control and an overall lack of public health awareness and response, although it is likely that other circumstances, including environmental, social and virological factors, contributed as well.

The first report of dengue disease incidence in the highlands of Nepal was during the 2010 outbreak [17]. In 2016, 3.12% of the reported cases of dengue were from highland regions, with 0.4% from the city of Kathmandu. Kathmandu is by far the largest urban area of Nepal, with around 4 million inhabitants as of 2016 [21]. Nepal is a poor country and Kathmandu especially has a high level of poverty featuring a considerable amount of sub-standard housing and water hygiene. The resource-poor health, social and economic infrastructures of Nepal in general and Kathmandu in particular, along with an increasing average temperature, are likely among the factors leading to the increased spread and incidence of DENV and dengue disease into highland areas. Although the 2006 dengue outbreak consisted of a relatively low number of reported cases of disease, all four DENV serotypes were detected [10]. However, the 2010 and 2013 dengue epidemics each featured a single dominate serotype, DENV-1 and DENV-2, respectively [18]. The occurrence over the past 12 years of multiple circulating serotypes, along with an overall increasing burden of dengue disease cases, suggests that the number of severe dengue disease cases is likely to rise in Nepal in the coming years [18]. Similar epidemiologic trends, in which ADE associated with multiple circulating serotypes has been considered a significant factor, have been observed in other regions such as in Indonesia and Brazil [22, 23]. Circulation of DENV-1 was also documented during 2014–2016 in several countries neighboring Nepal including India, China, Pakistan, Bangladesh and Sri Lanka [24–28].

### Conclusion

This report offers a clinical and epidemiological profile of the most recent major dengue outbreak in Nepal. Our documentation of the geographic incidence, symptomology and serotype association of the 2016 dengue outbreak will hopefully be useful to Nepalese government agencies to help strengthen mosquito surveillance and control as well as DENV vaccination, disease diagnosis and treatment programs.

## Limitation

Authors could not performed sequencing of the isolated dengue virus because of limited budget.

## Additional file

**Additional file 1: Table S1.** Primers used for DENV RTPCR and serotype-specific PCR.

## Abbreviations

DF: dengue fever
DHF: dengue hemorrhagic fever
DSS: dengue shock syndrome
DENV-1: dengue virus serotype-1
DENV-2: dengue virus serotype-2
DENV-3: dengue virus serotype-3
DOHs: Department of Health Services
EDCD: Epidemiology and Disease Control Division
ELISA: Enzyme Linked immunosorbent Assay
NPHL: Nepal Public Health Laboratory
PCR: polymerase chain reaction
RDT: rapid diagnostic kit
TU: Tribhuvan University
