# Supplementary material for: Profile of the 2016 dengue outbreak in Nepal
Source: BMC Res Notes. 2018 Jul 3;11:423. doi: 10.1186/s13104-018-3514-3 (PMC6029055; doi:10.1186/s13104-018-3514-3)
Supplement: Supplementary file 1 — Additional file 1: Table S1. Primers used for DENV RTPCR and serotype-specific PCR. [file 13104_2018_3514_MOESM1_ESM.docx]

**Table S1:**

| **Primer name** | **Sequence (5’ – 3’)** | **Location in DENV reference sequence** | **Reference Sequence (GenBank Accession No.)** |
| --- | --- | --- | --- |
| **D1F** | TCAATATGCTGAACGCGCGAGAAACCG | 134 - 161 | AF038403 |
| **DencomR2** | GCNCCTTCDGMNGACATCC | 785 - 767 | AF038403 |
| **NTS1** | CTGGTTCCGTCTCAGTGATCCGGGGG | 620 – 595 | NC_001477 |
| **NTS2** | AACGCCACAAGGGCCATGAACA | 254 – 233 | AY858036 |
| **NTS3** | TGCTGGTAACATCATCATGAGACAGAGCG | 427 – 399 | NC_001475 |
| **NDen4** | CTCTGTTGTCTTAAACAAGAGAGGTC | 527 - 502 | NC_002640 |
